# Prognosis of patients with apparent treatment-resistant hypertension—a feasibility study

**DOI:** 10.1186/s40814-018-0232-5

**Published:** 2018-01-30

**Authors:** Peter Hayes, Hannah Kielty, Monica Casey, Liam G. Glynn, Gerard J. Molloy, Hannah Durand, John Newell, Andrew W. Murphy

**Affiliations:** 1 Discipline of General Practice, School of Medicine, National University of Ireland, Galway, Ireland; 2 School of Medicine, NUI Galway, Galway, Ireland; 3 Graduate Entry Medical School, University of Limerick, Limerick, Ireland; 4 School of Psychology, NUI Galway, Galway, Ireland; 5 School of Mathematics, Statistics and Applied Mathematics, NUI Galway, Galway, Ireland; 6 Health Research Board Primary Care Clinical Trials Network, Galway, Ireland

**Keywords:** Hypertension, Primary care, Treatment resistant hypertension, Prognosis, Feasibility

## Abstract

**Background:**

Most cases of hypertension can be effectively treated with lifestyle changes together with medications, but within this population lies a group with more difficult to treat hypertension—those with apparent treatment-resistant hypertension (aTRH). The American Heart Association and the UK National Institute for Health and Care Excellence have both highlighted the need for further research into the prognosis of patients with resistant hypertension, both apparent and true.

**Methods:**

In 16 practices affiliated to a university research network, 646 patients had been identified with apparent treatment-resistant hypertension. To inform a planned full cohort study of these patients, we conducted a feasibility study within three practices to determine participation of practices and patients, availability of outcome measures and data collection times.

**Results:**

All three practices fully participated and 205/210 (98%) patients were followed up for a median of 23 months. Thirty-five outcome events of interest occurred—the most common was the new onset of retinopathy (9 cases). Eight percent (17/210) had the main composite outcome of death or serious incident cardiovascular event. Of the six patients who died, identification of cause of death was possible from practice records in five; the national General Register Office was successfully used for the final patient. There were 123 admissions, both day and overnight, recorded in 94 individual patients. Average manual systolic blood pressure measurements improved from baseline by 5 mmHg to 138 (SD 19) mmHg; diastolic remained the same at 75 (SD 12) mmHg. Average eGFR increased from 58.8 (SD17.4) to 66 (SD19.7) mls/min/1.73m^2^. The average time for data collection per patient was 12 mins.

**Conclusions:**

This study demonstrates that the proposed methodology for a full cohort study within general practice of patients with apparent treatment hypertension is both acceptable to practices and feasible. An adequately powered subsequent follow-up study of the entire cohort appears possible.

## Background

Arterial hypertension accounts for, or contributes to, 62% of all strokes and 49% of all cases of heart disease [1]. Most cases of hypertension can be effectively treated with lifestyle changes and/or medications, but within this population lies a group with more difficult to treat hypertension—those with treatment-resistant hypertension (TRH). TRH is defined as high blood pressure in a patient taking three or more differing groups of anti-hypertensive medications (one of which must be a diuretic type medication) or any patient who is taking four or more anti-hypertensive medications irrespective of blood pressure level [2]. Doses should be the optimal tolerated doses for each particular medication, lifestyle issues should also be addressed and white coat hypertension needs to be examined by ambulatory blood pressure monitoring (ABPM). When issues of dosing, medication adherence and white coat hypertension have not yet been ruled out, the term apparent treatment-resistant hypertension (aTRH) is utilised.

A recent meta-analysis of the prevalence of aTRH for a total population of 961,035 patients with diagnosed hypertension yielded prevalences for observational studies and trials respectively of 13.7 and 16.3% [3]. Individual study prevalence varied between 4.2 and 25.4%. Having established that resistant hypertension is apparently common, a key next step is to determine how important it is. Multiple cross-sectional studies comparing patients with aTRH to those without have suggested increased frequency of target organ damage and later cardiovascular complications [4]. In a key study, Daugherty [5] provided the best evidence to date with an outcome-based study on the longitudinal assessment of a large cohort of patients with aTRH (*n* = 3960). She found a 50% increase in cardiovascular events (largely attributable to development of chronic kidney disease) compared to those whose blood pressure was controlled on three medications. This impressive study is however a retrospective study of electronic insurance data with no assessment of ABPM or dosing and limited assessment of adherence. Sim also confirmed a possible increased cardiovascular risk associated with aTRH in 60,327 patients [6]. Patients with resistant hypertension, compared to those without, had significantly increased hazard ratios for stroke and mortality, respectively, of 1.14 (95% CI 1.10–1.19) and 1.06 (95% CI 1.03–1.08). However, Irvin [7] in retrospectively comparing similar groups found no significant increased risk of stroke but an increased risk for mortality. These studies, utilising large electronic databases, had large patient numbers but limited assessment of adherence, dosing and white coat hypertension. The American Heart Association [8] and the UK National Institute for Health and Care Excellence [9] have both highlighted the need for further high-quality prospective research into the prognosis of patients with resistant hypertension.

Previously, in a cross-sectional study of 6691 patients with hypertension in 16 Irish general practices, we identified 646 patients in Irish general practice with aTRH, whose files were individually reviewed and in whom pseudo-resistance was also examined [10]. These 646 patients therefore represent a unique general practice cohort of patients with comprehensive assessment for true treatment-resistant hypertension. It is planned to conduct a future prognostic cohort study of these 6691patients to compare those with apparent treatment-resistant hypertension and those with essential hypertension. In anticipation of this, we conducted a feasibility study to determine participation of practices and patients, availability and frequency of outcome measures and data collection times required.

## Methods

### Design and setting

The original cross-sectional study has been comprehensively described elsewhere [10]. In short,16 general practices in the university-affiliated research network WestREN, representative of the Irish population, participated [11]. All used the same practice software system (Socrates®) and the International Classification for Primary Care (ICPC-2) coding of chronic diseases. Ireland does not have universal registration with a general practitioner. All patients aged over 70 years, or below 6 years, and those below defined income levels receive free general practitioner care. These patients represent almost 40% of the general population and are registered with the Primary Care Reimbursement Service (PCRS; http://www.hse.ie/eng/staff/PCRS/) with the remainder being described as private patients and able to see any general practitioner. We therefore included in the cross-sectional sample all PCRS patients and those private patients who had attended the practice in the last year.

Each practice ran a standard ATC drug search identifying patients on any possible hypertensive medications as defined by the British National Formulary 69th Edition (https://www.amazon.co.uk/British-National-Formulary-BNF-69/dp/0857111566). Two researchers (PH and MC), in conjunction with the practices, then reviewed the record of each individual patient who was reported as being on one or more hypertensive medications and determined if they were hypertensive or not, had a previous ambulatory blood pressure measurement or not and what hypertensive medications and doses they were currently receiving. This work fulfilled for the general practitioners, the Irish Medical Council requirement, to conduct an annual audit.

Patients were then identified as being hypertensive if this diagnosis was recorded in clinical notes by their GP or if they had the appropriate diagnosis code recorded in the patient file (i.e. International Classification of Primary Care codes for hypertension-K87, K87). No start date for the diagnosis was recorded; this is not therefore an incident cohort.

### Recruitment

For this feasibility study, three practices were pragmatically chosen from the original 16 to reflect practice diversity—one was small and rural, one a medium and rural practice and one a large and mixed urban-rural practice. All 16 practices used the same practice software system (Socrates®) to store patient data, were located within 1-h travel of the university and had a mix of PCRS and private patients.

There were no incentives provided to these practices to participate; however, a stipend of €1000 was paid to all 16 practices when assembling the initial cohort as an acknowledgement for the extra workload involved. All 210 patients in these three practices were eligible to participate.

### Data collection

In each of the three practices, a manual review of each patient’s individual patient electronic record was performed by two trained researchers (HK and MC).

Both researchers participated in seminars on resistant hypertension. Training was also provided on how to use the practice software system (Socrates®) and more specifically on how to search for the data required. Both researchers worked in one practice together, and then, HK completed the other two practices, recording any issues for later clarification.

### Outcomes

We recorded the time spent in each practice for data collection, the proportions of patients whose records could be successfully accessed and who had an outcome of interest.

The proposed main composite outcome measurement is the same as that used by Daugherty [5]: all-cause mortality and incident cardiovascular events which include nonfatal myocardial infarction, heart failure, stroke or chronic kidney disease (CKD). Identification of cause of death was primarily through hospital or physician notices in patient’s files or through death certs. Where these were not informative, the national online General Register Office was utilised https://www.welfare.ie/en/Pages/Apply-for-Certificates.aspx.

Identification of the specified conditions are based on diagnosis codes, hospital correspondence and lab data. Patients are described as having diabetes if they have ICPC codes T89 (diabetes insulin-dependent) or T90 (diabetes non-insulin-dependent) or are taking insulin or oral hypoglycaemic agents. Patients are described as having CKD if an estimated glomerular filtration rate (eGFR) less than 60 mls/min/1.73m^2^ was recorded. Patients are described as having cardiac failure if they have ICPC code K77 or are noted to have this condition on hospital correspondence.

Recent blood pressure (BP) readings (manual and 24-h ambulatory) were also recorded, as was anti-hypertensive medication and doses. Hospital admissions and diagnoses were identified by discharge summaries. Day admissions largely involved medical or surgical assessment unit visits, angiograms or specialised testing requiring a ward admission. We did not include patients presenting for simpler tests such as echocardiograms or stress tests as a day admission.

### Power calculation

Table 3 illustrates the outcome events for patients with apparent treatment-resistant hypertension over a median of 1.9 years. Daugherty et al., for their main composite outcome over 3.8 years, found that 18 and 13.5% of patients with apparent treatment hypertension and simple hypertension respectively had the outcome. 8.3% of our cohort had the equivalent of Daugherty’s composite outcome and 17% a combination of all cardiovascular events. This suggests that a review of our entire original cohort over 4 years will result, at least, in similar outcome proportions to those of Daugherty.

Taking our original cohort of 6045 patients with simple hypertension and 646 patients with aTRH, allowing for a loss to follow-up of 10% and applying the proportions of 18 and 13.5%, with a significance level of 0.05, confirms that the cohort has 80% power to show a significant difference.

### Statistics analysis plan

The primary response is the probability of patients developing the outcome of interest as defined by Daugherty. A logistic regression model will be used to compare the probabilities between the two groups while adjusting for explanatory variables such as age, gender, socioeconomic status, baseline blood pressure, presence of diabetes or kidney disease as appropriate. Initially all explanatory variables will be included as adjusters, and the ridge regression and the LASSO [12] will be used to account for any multicollinearity present among the explanatory variables. Classification trees will be used to identify potentially useful interactions.

## Results

**Fig. 1:**
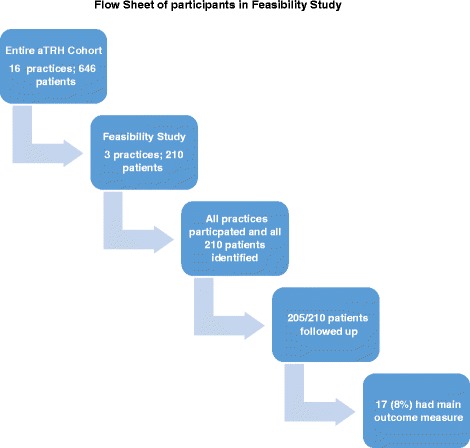
Flow sheet of participants in the feasibility study

All three contacted practices agreed to participate. Of the original 210 patients in these practices, data was available for 205 patients (97.6%). Five patients had transferred practice and data was not available (see Fig. 1). Follow-up was for a median of 23 months. File search took place from June 19 to July 14 2017—a total of 4 weeks for three practices. The total time spent in data collection at the practices was 44 h which was an average of 12.6 min per patient.

Table 1 shows the characteristics of the original cohort (*n* = 646), and Table 2 illustrates the characteristics of the 205 patients examined for prognosis. The average manual BP reading was 138 mmHg (SD19) systolic blood pressure and 75 mmHg (SD12) diastolic blood pressure. A mean reduction of 5 mm Hg systolic (SD11mmHg) is seen between the latest clinic readings and the original cohort's readings. One hundred twelve patients (over the previous 23 months median follow-up period) had ABPM, and the daytime average ABPM reading was 136 (SD16)/74 (SD10) mmHg. There were anti-hypertensive medications and or dose changes in 57%. Nine percent (*n* = 18) no longer met the minimum inclusion criteria for aTRH of three or more medications. Average eGFR increased from 58.8 (SD17.4) to 66 (SD19.7) mls/min/1.73m^2^. From the 210 patients examined, 65 persons have true resistant TRH (26%) and 140 persons have pseudo-resistant TRH (Table 2), while from the main cohort of 646 persons with aTRH, 30% (*n* = 166) have true TRH and 70% (*n* = 480) have pseudo-resistant TRH (Table 1).

**Table 1:** Demographic and clinical details for original cross sectional group (*n* = 646)

Demographic	(Mean if applicable)	Standard deviation (from mean)
Age (years)	71.1	12
Sex	54% male	
Eligible for Primary Care Reimbursement Scheme (marker of household income)	82% (*n* = 530)	
Clinical details		
Diabetes	37% (*n* = 237)	
CKD	40% (*n* = 258)	
Cardiac failure	12% (*n* = 79)	
Manual BP	142/78 mmHg	18/12 mmHg
Recent ABPM BP (where available within 6 months)	148/81 mmHg (*n* = 74)	21/14 mmHg
Medications		
No. of anti-hypertensive drugs used	3.7	0.7
4 or more anti-hypertensive drugs	56% (*n* = 364)	
Sub-divisions		
True TRH	166	
Pseudo-resistant TRH	480

**Table 2:** Demographic and clinical details for patients with apparent treatment-resistant hypertension in feasibility study (*n* = 210)

Demographic	(Mean if applicable)	Standard deviation (from mean)
Age (years)	73.7 YOA	11.2
Sex	56% male	
Eligible for Primary Care Reimbursement Scheme (marker of household income)	80% (*n* = 167)	
Clinical details		
Diabetes	31% (*n* = 65)	
CKD	41% (*n* = 87)	
Cardiac failure	15% (*n* = 31)	
Manual BP	138/75 mmHg	19/12 mmHg
Recent ABPM BP (where available within 23 months median follow-up period)	134/76 mmHg (*n* = 112)	16/10 mmHg
Medications		
No. of anti-hypertensive drugs used	3.7	1.06
4 or more anti-hypertensive drugs	51%	
Subdivisions		
True TRH	26% (n = 65)	
Pseudo-resistant THR	74% (*n* = 140)	

Thirty-five events of interest occurred—the most common was the new-onset retinopathy at 4.3% (9/210) (see Table 3). Of the six patients who died, identification of cause of death was possible from practice records in five; the national General Register Office was successfully used for the final patient. Seventeen patients from 210 in the examined cohort (8%) had the main composite outcome of death or an incident cardiovascular event of nonfatal myocardial infarction, heart failure, stroke or CKD. There were 123 admissions recorded in 94 individual patients, both day and overnight, and these are detailed in Table 4.

**Table 3:** Outcome events for patients with apparent treatment-resistant hypertension in feasibility study (*n* = 205)

Any cause of death^a^	6
CVD cause death	1
New non-fatal myocardial infarction^a^	2
New non-fatal congestive cardiac failure^a^	5
New non-fatal cerebrovascular accident^a^	3
New-onset chronic kidney disease^a^	1
New non-fatal transient ischaemic attack	5
New-onset retinopathy	9
New atrial fibrillation	3
New onset peripheral arterial disease	0
Total	
Number of events total	*35*
Number of individuals with composite outcome	*17*

^a^These are included in the main outcome composite outcome (Daugherty)

**Table 4:** Admissions for patients with apparent treatment-resistant hypertension in feasibility study (123 events, 94 patients)

Admissions	Number (*n* = 123)	Percentage of total
Non-CVD hospitalisations	62	50% (62/123)
Non-CVD day admissions	27	22% (27/123)
CVD hospitalisations	23	19% (23/123)
CVD day admissions	11	9% (11/123)

## Discussion

Prior to conducting a larger cohort study of all 646 patients in all 16 practices, we needed to demonstrate the feasibility of the proposed methodology by addressing key uncertainties. Our key uncertainties related to the acceptability of follow-up to practices, the availability of outcome data (especially in a health system without universal registration) and the workload involved. By explicitly addressing these uncertainties in a feasibility study, we can confirm that an adequately powered subsequent follow-up study of the entire cohort is possible.

We attempted to ensure that the follow-up strategy imposed little burden on practices, and this appeared to work well. The average research time of 12 min per patient includes data collection only, but travel to practices was a mean of 80 min return (maximum trip—2 h return). The main variation in data collection time between practices was the number of patient files examined. The main variation in data collection time between individual patients was the number of recorded health care interactions.

Tips for ensuring a smooth research process include pre-booking time slots for practice computers, showing flexibility regarding access times; multiple half days may be available as opposed to full days and administrator/front desk workload acknowledgement, e.g. teas, courtesy and thank you cards. The signing of individual practice confidentiality agreements was also seen as important. These may not be required by local ethics committees, but GP’s data protection fears are eased by such. Tips for ensuring accurate data collection include ensuring the adequate training of researchers in use of the relevant patient’s data management system and meeting with practice administrators prior to data collection. Administrators will know where specific data items are stored, e.g. ABPM reports and how the files of those who are deceased or have moved on are managed in individual practices. These issues will need to be factored into future planning.

All practices that participated were part of a university-affiliated research network WestREN. Regular e-mail updates and annual meetings where research projects were discussed are key to maintaining practice participation. Allowing practices to fulfil their mandatory audit commitments, while participating in research projects, is also welcome. The very low numbers lost to follow-up and completeness of data are pleasing; however, this may not be replicated in the larger level, and due allowance has been made in the power calculation.

Outcome measurement data appeared readily available. Coding of individual consultations is not common in Irish general practice. A combination of coding for the key diagnoses of interest by practices, together with review of scanned written hospital discharges, while time-consuming, appeared to overcome this challenge. In the last 5 years, public hospitals in the region have introduced electronic discharge summaries which automatically enter the individual patient electronic record, significantly aiding the outcome assessment process. Identification of cause of death from practice data also appeared straightforward with only one of six deaths requiring access to the online State Register. This was needed for an in-hospital patient death, where cause of death was unclear from practice clinical notes and notice of death, as opposed to cause of death, was recorded.

Two outcome measurements were of particular interest. New-onset retinopathy has not been reported in other similar cohort studies. It was our most common outcome event. With over a third of our patients with apparent resistant hypertension having diabetes, this is of potential significance. Consideration may need to be given to including retinopathy in any composite outcome measurement. It highlights the importance of the work of the COMET (Core Outcomes Measures in Effectiveness Trials) group who are agreeing standardised sets of outcomes (http://www.comet-initiative.org). There is, as yet, no agreed core outcome set in the study of resistant hypertension. Contrary to our expectations, renal function as estimated by eGFR, improved rather than deteriorated over the duration of follow-up. Clearly, our sample size limits the interpretation of this. However, it does emphasise the need for serial eGFR measurements to be utilised rather than ‘once off’ recordings.

### Comparison with other work

Table 5 illustrates the key parameters of previous studies reviewing the prognosis of patients with aTRH. Our study is distinguishable as both the only study based in general practice and the only study to have individual patient record review. This facilitates consideration of dosing, adherence and white coat hypertension which are often overlooked in other studies. Similar to Daugherty, we will be able to, with adequate power, compare outcomes for patients with simple hypertension to those with treatment-resistant hypertension (Table 2). However, the numbers with true resistant hypertension will be small making meaningful comparisons difficult.

**Table 5:** Comparison between prognostic studies of patients with apparent treatment-resistant hypertension

Study	Setting	Record	Duration of follow-up	Pseudo-resistance considered
Daugherty (2012) [5]	Primary/secondary care health insurance	Electronic database	Median 3.8 years	No
Sim et al. (2015) [6]	Primary/secondary care health insurance	Electronic database	Retrospective cohort 5 years	No
Irvin et al. (2014) [7]	Population-based cohort recruited	Patient interview	Median 4.4–6 years	No (adherence—yes)
Hayes et al. (planned 2020)	Primary care	Manual file search	Median 5 years	Yes

### Strengths and limitations

We based our methodology on a previous cohort study we conducted in the region which followed up 1605 patients with heart disease from 35 practices after 5 years [13]. This experience was invaluable in developing our protocol. The subsequent introduction of automatic hospital electronic discharges and enhanced coding by practices has aided cohort study conduct.

There are four significant limitations. Firstly, on balance, a non-random selection of practices was preferred as it allowed us to sample the diversity of practice size and location. We used three typical practices. It also facilitated transport issues for researchers, within a short available time period, to be taken into account. Other practices may have specific issues which we have not encountered, but the recording of electronic health care data is similar in the Socrates® practice software system, which is reassuring.

Secondly, the Irish health system is a complex amalgam of private and public providers. Other similar studies (Daugherty [5], Irvin [7]) had the significant benefits of an integrated health system facilitating collection of primary, secondary and tertiary care data. We can never be sure that some important outcome data is not missing. Thirdly, we did not follow up the five patients who transferred practice. This may be the main challenge in the future and will likely involve locating patients who have moved practice. These patients may subsequently have their data now recorded in an alternative electronic format. Consideration in the full cohort study to providing resources to do this is appropriate. Finally, the cohort was assembled in a cross-sectional study and not as an incident cohort with no recorded date of diagnosis.

## Conclusions

This study demonstrates that the proposed methodology for a full cohort study within general practice of patients with apparent treatment hypertension is both acceptable to practices and feasible. An adequately powered subsequent follow-up study of the entire cohort appears possible.

## Abbreviations

ABPM: Ambulatory blood pressure monitoring
*ATC*: Anatomical therapeutic chemical
aTRH: Apparent treatment-resistant hypertension
BP: Blood pressure
CI: Confidence interval
CKD: Chronic kidney disease
eGFR: Estimated glomerular filtration rate
SD: Standard deviation
tTRH: True treatment-resistant hypertension
